# A geometric model of defensive peripersonal space

**DOI:** 10.1152/jn.00691.2015

**Published:** 2015-10-28

**Authors:** R. J. Bufacchi, M. Liang, L. D. Griffin, G. D. Iannetti

**Affiliations:** ^1^Department of Neuroscience, Physiology and Pharmacology, University College London, London, United Kingdom;; ^2^Centre for Mathematics and Physics in the Life Sciences and Experimental Biology (CoMPLEX), University College London, London, United Kingdom;; ^3^School of Medical Imaging, Tianjin Medical University, Tianjin, People's Republic of China; and; ^4^Department of Computer Science, University College London, London, United Kingdom

**Keywords:** body, defense, personal space, modeling, blink reflex

## Abstract

Potentially harmful stimuli occurring within the defensive peripersonal space (DPPS), a protective area surrounding the body, elicit stronger defensive reactions. The spatial features of the DPPS are poorly defined and limited to descriptive estimates of its extent along a single dimension. Here we postulated a family of geometric models of the DPPS, to address two important questions with respect to its spatial features: What is its fine-grained topography? How does the nervous system represent the body area to be defended? As a measure of the DPPS, we used the strength of the defensive blink reflex elicited by electrical stimulation of the hand (hand-blink reflex, HBR), which is reliably modulated by the position of the stimulated hand in egocentric coordinates. We tested the goodness of fit of the postulated models to HBR data from six experiments in which we systematically explored the HBR modulation by hand position in both head-centered and body-centered coordinates. The best-fitting model indicated that *1*) the nervous system's representation of the body area defended by the HBR can be approximated by a half-ellipsoid centered on the face and *2*) the DPPS extending from this area has the shape of a bubble elongated along the vertical axis. Finally, the empirical observation that the HBR is modulated by hand position in head-centered coordinates indicates that the DPPS is anchored to the face. The modeling approach described in this article can be generalized to describe the spatial modulation of any defensive response.

the defensive peripersonal space (DPPS) is a portion of space surrounding the body characterized by a protective function (Cooke and Graziano 2003; de Vignemont and Iannetti 2015). Potentially harmful stimuli located within this space elicit stronger defensive reactions than stimuli located outside of it (Graziano and Cooke 2006). The DPPS surrounding the head has recently been identified in humans by recording the enhancement of the hand-blink reflex (HBR) when the stimulated hand is located close to the face (Sambo et al. 2012b). The HBR consists of the stimulus-evoked contraction of the orbicularis oculi muscles, measured by recording their activity with surface electromyography (EMG). The HBR magnitude is typically estimated by integrating the EMG activity (area under curve, AUC).

Compared with other physiological measures, the HBR is ideal to investigate the spatial features of the DPPS, for two main reasons. First, in a Sherringtonian sense the HBR has a purely defensive value. Second, a change of the position of the stimulated hand in egocentric coordinates does not alter the intensity of the sensory input eliciting the response. The use of the blink reflex elicited by, for example, auditory stimuli would present the major drawback of different stimulus intensities when the stimulus is in different spatial locations.

The enhancement of the HBR by hand proximity results from a tonic and selective top-down modulation of the excitability of the brain stem interneurons mediating the HBR. This modulation is finely adjusted to ensure appropriate behavior, depending on high-level contextual factors, like the probability of stimulus occurrence and the presence of defensive objects close to the face (Sambo et al. 2012a).

Examining the HBR enhancement as a function of the position of the eliciting stimulus in external space coordinates has allowed a preliminary characterization of the spatial features of the DPPS (Sambo and Iannetti 2013). Indeed, it has been shown that *1*) HBR magnitude does not linearly increase with the proximity between the stimulated hand and the eye, suggesting that the DPPS has relatively sharp boundaries, and *2*) there are clear interindividual differences in its extension related to individual levels of anxiety.

However, in Sambo and Iannetti (2013) the position of the threatening stimulus in external space was only modulated across four positions along a single axis perpendicular to the face, at a downward angle of ∼15° from the eye (Fig. 1). Furthermore, the estimate of the individual DPPS shape was obtained with step models, in which there is no information about HBR strength in between the four stimulation positions. Consequently, this modeling approach does not allow making any predictions about the HBR response elicited by stimuli in spatial locations where no measurements were taken and thus cannot be used to derive a finer shape of the DPPS surrounding the face.

**Fig. 1.**
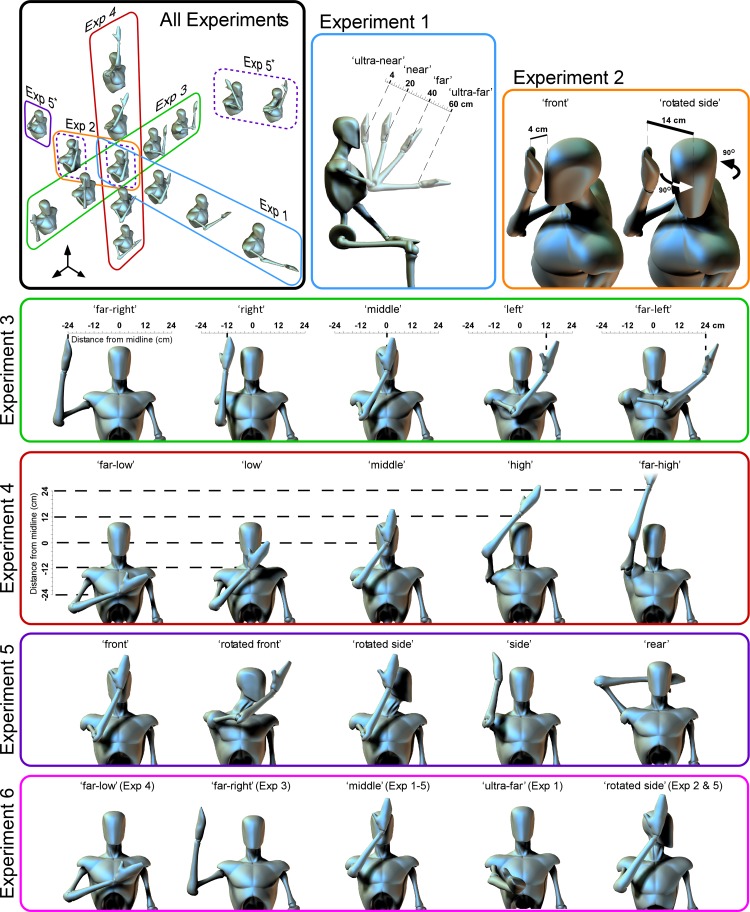
Hand positions at which the hand-blink reflex (HBR) was recorded in the 6 experiments. The HBR was elicited by electrical stimulation of the right median nerve at the wrist. In *experiment 1*, the hand was placed 4, 20, 40, and 60 cm from the face along the sagittal plane at a downward angle of 15° from the eye (Sambo and Iannetti 2013). In *experiment 2*, the “front” position was the same as the nearest position of *experiment 1*, and the “rotated side” position was the same as “front” but with the head rotated 90° to the left (Sambo et al. 2012b). In *experiment 3*, the hand was placed in 5 positions along a coronal plane 4 cm in front of the face at eye height. In *experiment 4*, the hand was similarly placed at 5 positions on the same coronal plane but along a vertical line on the body midline. In *experiment 5*, the hand was placed in 3 positions in head-centered coordinates (in front of the head, beside the head, and behind the head) while the head either faced straight ahead or was rotated by 90°. Therefore, in head-centered coordinates, 2 pairs of these conditions (“side” and “rotated side”; “front” and “rotated front”; indicated by dashed boxes in the *top left*) were identical. In *experiment 6*, the hand was placed in 5 positions, each of which was present in at least 1 of *experiments 1–5*. *Top left*: an overall view of the postures used in all experiments.

Therefore, in the present work we aimed to obtain a general characterization of the spatial features of the DPPS by formulating a family of geometric models. These models simulate how the brain computes the different levels of threat represented by identical stimuli that differ in their egocentric spatial position. In these models, the strength of the defensive response is expressed as a mathematical function of the spatial location of the threat. To formulate and test the models, we analyzed data from two previously published experiments (Sambo et al. 2012a, 2012b) and four new experiments (Fig. 1). In two of the new experiments, the position of the threat was modulated along the medio-lateral and rostro-caudal axes on a coronal plane in front of the face. In the third new experiment, the threat was placed in front of, to the side of, or behind the head, while the head orientation was either forward or sideways. The fourth new experiment included a subset of the stimulation positions used in all other experiments, to control for the variability in response magnitude across data sets and therefore allow for a combined analysis of all data sets. We simultaneously fitted the data from these six experiments to a series of geometric models in which the HBR magnitude was calculated from the geometric probability of the individual being hit by a threat and which covered the entirety of space surrounding the body rather than just the positions at which the HBR magnitude was measured.

Besides the position of the threat, another important factor determining the goodness of fit (GoF) of the geometric model is the shape of the body district to be defended. Therefore, we also tested whether and how a range of shapes of the defended area altered the GoF. Theoretically, the number of testable shapes is infinite. However, a large number of such shapes are physiologically implausible. Therefore, and given that the HBR is a defensive response performed by muscles innervated by the facial nerve, we limited the testing to four different geometries representing defended areas related to the head: the entire head (full ellipsoid), the face (half-ellipsoid), the top part of the face (quarter-ellipsoid), or the eyes (two half-ellipsoids) (Fig. 2). Thereby, we assessed how the brain geometrically represents the body part to be defended.

**Fig. 2.**
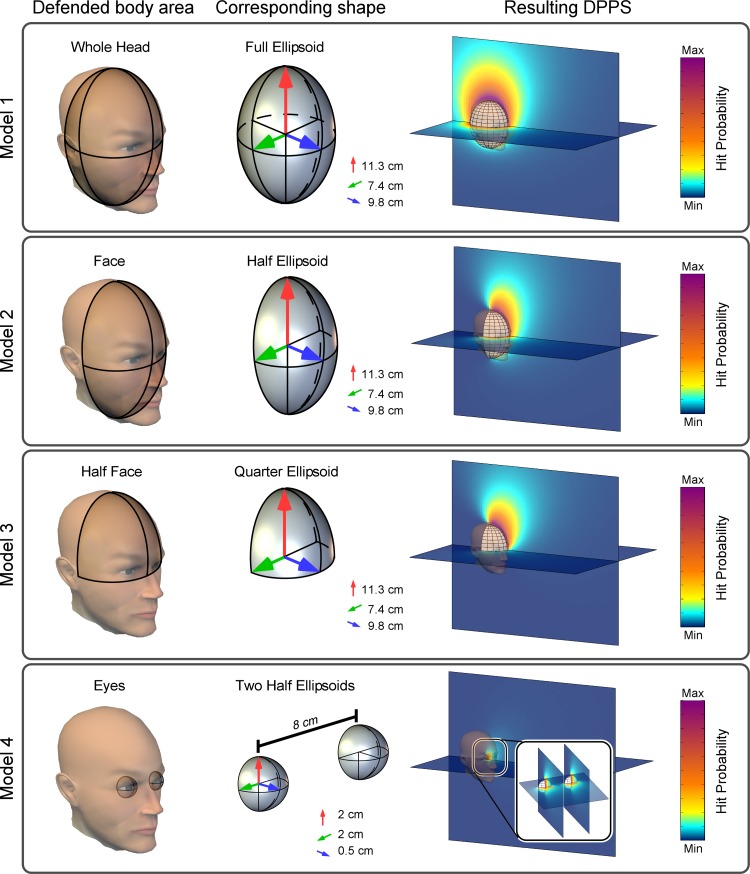
Geometric models. In these models the increase in HBR magnitude reflects the probability of a defended area being hit. Here we consider 4 possible shapes of defended areas, corresponding (from *top* to *bottom*) to the entire head, the face, the half-face, and the eyes. These areas are approximated by an entire ellipsoid, a half-ellipsoid, a quarter-ellipsoid, and 2 smaller half-ellipsoids, respectively. 3D plots on *right* show the predicted HBR enhancement depending on the spatial location of the stimulus. DPPS, defensive peripersonal space.

## MATERIALS AND METHODS

### 

#### Participants.

We analyzed the data collected in two previous experiments (*experiments 1* and *2*) as well as data from four new experiments (*experiments 3*, *4*, *5*, and *6*). All experiments were conducted in groups of healthy participants, as follows: *experiment 1*: 15 participants (7 women, 8 men), age range 20–37 yr, mean age ±SD 27.4 ± 5.7 yr (Sambo and Iannetti 2013); *experiment 2*: 7 participants (3 women, 4 men), 26–37 yr, 32.9 ± 5.7 yr (Sambo et al. 2012b); *experiment 3*: 10 participants (7 women, 3 men), 18–40 yr, 24.1 ± 6.7 yr; *experiment 4*: 10 participants (7 women, 3 men), 21–30 yr, 22.7 ± 3.9 yr; *experiment 5*: 10 participants (5 women, 5 men), 18–31 yr, 22.9 ± 3.4 yr; and *experiment 6*: 11 participants (6 women, 5 men), 19–40 yr, 24.0 ± 5.7 yr. The 41 participants in the four new experiments (*3–6*) were HBR responders (Miwa et al. 1998) identified from a group of 63 recruited subjects. Therefore, 65% of recruited subjects were HBR responders. This figure is consistent with previous reports (Miwa et al. 1998; Sambo et al. 2012a, 2012b; Sambo and Iannetti 2013). Importantly, this figure is consequent to the large variability in HBR threshold among the population and reflects the response elicited by stimulus intensities within ethically acceptable stimulation energies. Therefore, the results observed in these HBR responders are likely to generalize to the entire population.

All participants were right-handed. Participants gave written informed consent before taking part in the study. All procedures were approved by the local ethics committee.

#### Stimulation and recording.

Details of stimulation and recording procedures of *experiments 1* and *2* are reported in Sambo and Iannetti (2013) and Sambo et al. (2012a), respectively. The procedures for *experiments 3–6* were identical, except for the hand positions, as detailed below and summarized in Fig. 1. Briefly, transcutaneous electrical stimuli were delivered to the median nerve at the wrist. In each participant, we first determined the stimulus intensity able to elicit a well-defined and stable blink reflex. This was achieved by increasing the stimulus intensity until a clear HBR was observed in three consecutive trials or the participant refused a further increase of stimulus intensity (Valls-Solé et al. 1997). Participants were told that the stimulation would elicit a strong, unpleasant, but not painful sensation. Accordingly, none of the participants reported painful sensations, even at high stimulus intensities.

The mean stimulus intensities used in the experiments were as follows: 42.3 mA (*experiment 1*), 43.5 mA (*experiment 2*), 53.8 mA (*experiment 3*), 48.2 mA (*experiment 4*), 39.0 mA (*experiment 5*), and 55.8 mA (*experiment 6*). The stimulus duration was 200 μs, and the interval between successive stimuli was ∼30 s. EMG activity was recorded from the orbicularis oculi muscle, bilaterally, with surface Ag-AgCl electrodes. Signals were amplified and digitized at a sampling rate of 8,192 Hz.

In *experiment 1* we recorded HBR responses while the participants' stimulated hand was placed at four distances from their eyes: “ultra-far”: 60 cm; “far”: 40 cm; “near”: 20 cm; “ultra-near”: 4 cm. The hand not undergoing the postural manipulation was never stimulated and was kept on a table throughout the duration of the experiment. During the recording participants were instructed to keep their gaze on a fixation cross (1.5 × 1.5 cm) placed at ∼30 cm from the eyes and 45° below eye level.

The experiment consisted of two blocks. In each block, stimuli were delivered to either the right or the left wrist (i.e., the wrist of the arm undergoing the postural manipulation). The order of blocks was balanced across participants. In each block, 32 electrical stimuli were delivered: 8 for each of the 4 hand-face distances. The data for the two blocks were pooled, resulting in a total of 16 stimuli per condition. The stimuli were delivered in pseudorandom order, with the constraint that no more than two consecutive stimuli were delivered for the same hand-face distance.

In *experiment 2* we recorded the HBR while the position of both the hand and the arm was kept constant and the proximity of the stimulated hand to the face was manipulated by rotating the head. Thus the participants' forearm was kept flexed in the same near position all the time and their head was either kept straight in anatomical position (“front” condition) or rotated sideways by 90° (“rotated side” condition).

This experiment also consisted of two blocks. In each block, 20 stimuli were delivered to either the right or the left wrist. Of these, 10 were delivered in the front condition and 10 in the side condition in alternating trials. The order of the blocks was balanced across participants.

In *experiment 3* we recorded the HBR while the participants' right hand was placed in five positions on a coronal plane located 4 cm from the nose, along a horizontal line at eye level (Fig. 1). The five positions were symmetric with respect to the midline, as follows (negative values denote positions on the left side): “far-right”: −24 cm; “right”: −12 cm; “middle”: 0 cm; “left”: +12 cm; “far-left”: +24 cm. The positions were marked out on a board placed in front of the participant.

In *experiment 4* we recorded the HBR while the participants' right hand was placed in five positions on a coronal plane located 4 cm from the nose, along a vertical line on the body midline (Fig. 1). The five positions were symmetric with respect to eye level, as follows (negative values denote positions below eye level): “far-low”: −24 cm; “low”: −12 cm; “middle”: 0 cm; “high”: +12 cm; “far-high”: +24 cm. The positions were marked out on a board placed in front of the participant.

In *experiment 5* we recorded the HBR while both the position of the participants' right hand and the direction of the participants' face were manipulated. This yielded five different conditions. Two conditions were identical to *experiment 2* (“front” and “rotated side” conditions). In the third condition, the head was rotated 90° to the left and the hand was placed directly in front of the face (“rotated front” condition). In the fourth condition, the head faced straight forward and the hand was placed to the right of the head, beside the right ear (“side” condition). In the fifth condition, the head faced straight forward and the hand was placed directly behind the head (“rear” condition).

In *experiment 6* we recorded the HBR while the participants' right hand was placed in five positions, each of which was present in at least one of *experiments 1–5*. In the “middle” position the hand was placed on the midline at eye level, 4 cm from the nose. This position was included in all other experiments (and labeled as “ultra-near” in *experiment 1*, “front” in *experiments 2* and *5*, and “middle” in *experiments 3* and *4*). The other four positions were the “ultra-far,” “rotated side,” “far-right,” and “far-low” positions from *experiments 1*, *2*, *3*, and *4*, respectively.

As expected, in *experiments 1* and *2* there was no difference in the HBR elicited by stimulation of the right and left hands (*P* = 0.09 and *P* = 0.51, respectively; paired *t*-test). Therefore, in *experiments 3–6* we only stimulated the right hand, thereby being able to explore the effect of five different hand positions within a single experiment. *Experiments 3–6* consisted of two blocks. In each block, 25 stimuli were delivered to the right wrist, 5 at each hand position. Hence, across the two blocks, a total of 10 stimuli per hand position were delivered (50 stimuli across the 5 hand positions). This number of stimuli per hand position was chosen to obtain a reliable HBR, while still allowing us to explore as many hand positions as possible within one experiment. The order of hand positions was pseudorandomized, with the constraint that no more than two consecutive stimuli were delivered for the same hand position.

#### HBR magnitude normalization.

In all experiments the magnitude of the HBR was estimated as the AUC of each single-trial response, separately for each eye, as previously done (Sambo and Iannetti 2013). Within each trial, the AUCs of the response recorded from the eye ipsilateral and contralateral to the stimulated hand were subsequently averaged together to improve the response signal-to-noise ratio. The rationale for such a procedure was the evidence of no interaction between hand position and recording side in determining the HBR magnitude (Sambo et al. 2012a). The AUCs at each position were finally averaged across trials, to give a single average AUC value for each subject at each hand position.

To model the data sets from all six experiments and all subjects simultaneously, the AUCs were first shifted so that at the “ultra-near,” “middle,” and “front” positions (in, respectively: *experiments 1*; *3* and *4*; and *2* and *5*) the AUCs were set to 0 for each subject. Second, the AUCs in *experiments 1–5* were normalized by using the AUCs of the response at the corresponding positions in *experiment 6* as anchors. As an illustrative example, if the mean AUC at the “far-right” position in *experiment 3* was *X* and the mean AUC at that same “far-right” position in *experiment 6* was *Y*, given that the AUCs at the “middle” position of both experiments were set to 0, the AUCs of the responses in all positions of *experiment 3* were rescaled, as follows:

(1) $${\text{AUC}}_{\text{3},\text{RESCALED}}=\frac{Y}{X}{\text{AUC}}_{\text{3,ORIGINAL}}$$

#### Testing for normality and equal variance.

The validity of the considered models was assessed by their GoF to the mean HBR magnitudes at all positions. The GoF modeling approach compares the χ^2^-test statistic of the fit of any given model to the data to a χ^2^ distribution of the appropriate degrees of freedom, resulting in a GoF score and a corresponding *P* value. Therefore, if the GoF score is larger than 1.850 (which corresponds to the threshold of *P* = 0.05 in the χ^2^ distribution considered), the probability of the model being correct is smaller than 0.05, and the model must be rejected. Hence, the smaller the GoF score and the larger the *P* value of a model, the more strongly the model is accepted. This approach requires *1*) that data are normally distributed and *2*) that the variance across hand positions is equal. Because we fit the models to the mean HBR magnitudes at each position, we used the standard error of the mean as measure of data variance. The Anderson-Darling test was used to assess normality of the distribution and the Bartlett's test to assess differences of variance.

To meet these two requirements, data from two subjects in *experiment 1* had to be excluded. In these subjects the variance of the HBR response was different across hand positions (*P* = 0.005 and *P* = 0.0046; H_0_ = equal variance). After the exclusion of these subjects and power-transformation of the data (AUC → AUC^0.25^), HBR magnitude had equal variance (*P* = 0.3131; H_0_ = equal variance) and was normally distributed across all hand positions (Anderson-Darling test, *P* = 0.1319; H_0_ = normal distribution).

#### Modeling the defensive peripersonal space.

We characterized the DPPS with a series of geometric models.

These models use geometrically derived formulas to describe a danger function underlying the observed changes in HBR magnitude according to *1*) the position of the threat in external space and *2*) the spatial features of the area being defended.

This approach formalizes the intuitive idea that the closer a potentially harmful stimulus is to an individual, the greater its probability to do harm, which therefore results in a stronger defensive reaction as follows.

Consider an agent *A* and a threat *B*, which is potentially harmful to *A*. *A* can perform a defensive action called, in this context, a “blink.” We define as “hitting” the potential harm that *B* can do to *A*. We then assume the following: *1*) *B* is observed at a point in space connected to the center of *A* by the vector $\vec{r}$; *2*) as soon as *B* appears, it makes a linear hitting action; *3*) the hitting action occurs in a random direction; *4*) if the hitting trajectory intersects *A*, *A* is hit; *5*) *A* aims to minimize the probability of being harmed, which is proportional to the probability of being hit by *B*:

(2) $$P\left(B\;hits\;A,\overset{\to }{r}\right)$$

*6*) the purpose of a blink is to reduce the damage done by *B* if *A* is hit; *7*) a stronger blink will further reduce the damage caused by *B* to *A*; and *8*) blinking has a cost: the probability of *A* being harmed in other ways (besides being hit by *B*) increases with the strength of blinking. (If blinking had no cost, then *A* would never stop blinking at maximal intensity.)

Given these assumptions, the increase in blink strength should be related to *P*(*B* hits *A*,$\vec{r}$), as long as the probability of *A* being harmed in other ways (i.e., besides being hit by *B*) while blinking is smaller than the probability of being harmed by *B*. In other words, the agent *A* reacts to the threat *B* with a blink, whose magnitude is proportional to the probability of *A* being hit by *B*: *P*(*B* hits *A*,$\vec{r}$).

Therefore, the probability of being hit is reflected by the ratio between the number of trajectories of the hitting action that will result in *A* being hit and the number of all possible trajectories:

(3) $$P\left(B\;hits\;A,\overset{\to }{r}\right)=\frac{\Omega }{4\pi }$$

where Ω is the solid angle of agent *A* from the perspective of *B*, i.e., the portion of space within which a trajectory will result in *A* being hit, and 4π is the solid angle of all space. We calculated this ratio with a Monte-Carlo method, in which 2 million random trajectories originating from *B* were generated. Each trajectory was defined by a vector whose *x*, *y*, and *z* components were drawn from a standard normal distribution with unit variance. The effect of gravity on the trajectory of the threat was taken into account by a specific model parameter (*C*_grav_). This value was the shift of the mean of the normal distribution from which the *z*-components were drawn, and it was optimized to fit the data. As the number of trajectories approaches infinity, the ratio between the number of trajectories hitting *A* and the total number of trajectories approaches the probability of the agent *A* being hit (*Eq. 3*).

We linearly transformed the probability of *A* being hit *P*(*C*_grav_,**r**) into the magnitude of the defensive HBR response *S*(**r**), as follows:

(4) $$S(\overset{\to }{r})=aP(C_{grav},\overset{\to }{r})+b$$

where *a* and *b* are the two fitting parameters in the linear model. Therefore, *Eq. 4* allows the calculation of the blink strength at any point in space around the defended object.

Given the shape of the head, we considered four possible geometries for agent *A*. The first three geometries corresponded to *1*) the full head, *2*) the face, and *3*) the upper half of the face and were respectively modeled by a full ellipsoid, a half-ellipsoid, and a quarter-ellipsoid. The ellipsoid modeling these three geometries of agent *A* had semiaxes of 9.8 (*x*), 7.4 (*y*), and 11.3 (*z*) cm (Fig. 2). These values were derived from topometric atlases (United States Army 2000) and reflected the average dorso-ventral, medio-lateral, and rostro-caudal dimensions of the human head, respectively. The fourth geometry corresponded to *4*) the two eyes, and it was modeled by a pair of half-ellipsoids with semiaxes of 0.5 (*x*), 2 (*y*), and 2 (*z*) cm (Fig. 2). These values reflected the average dorso-ventral, medio-lateral, and rostro-caudal dimensions of the human orbit. For threat *B*, we consider a single geometry: a point.

For each considered shape of agent *A*, we optimized *a*, *b*, and *C*_grav_ in *Eq. 4*, to obtain a best fit of blink magnitude at each position for all six experiments simultaneously. Hence, three parameters were used to fit the models. The optimization was done by finding the minimum χ^2^-test statistic through the *fminsearch* function in MATLAB, which uses the Nelder-Mead simplex algorithm (Lagarias et al. 1998). This statistic also allowed us to calculate a GoF and its corresponding *P* value by comparing the χ^2^ statistic to a standard χ^2^ distribution.

## RESULTS

### 

#### Descriptive results.

To assess the overall effect of hand position on the HBR magnitude we performed one-way, repeated-measures ANOVAs (*experiments 1*, *3–6*), and a paired *t*-test (*experiment 2*). The factor “hand position” was a significant source of variance in all experiments (*experiment 1*: *F* = 28.0, *P* < 0.0005; *experiment 2*: *t* = −6.4, *P* = 0.001; *experiment 3*: *F* = 5.9, *P* = 0.018; *experiment 4*: *F* = 4.7, *P* = 0.042; *experiment 5*: *F* = 4.6, *P* = 0.004; *experiment 6*: *F* = 18.3, *P* < 0.0005). In *experiments 1*, *3*, *4*, *5*, and *6* we performed post hoc Tukey's tests to determine between which positions the HBR magnitudes differed.

In *experiment 1*, the HBR increased monotonically with the proximity between the stimulated hand and the face. There was a significant difference in HBR magnitude between all positions (*P* < 0.001), except between the “ultra-far” and “far” positions (*P* = 0.754).

In *experiment 3*, there was a significant difference between positions “middle” and “far-left” (*P* = 0.016), “middle” and “right” (*P* = 0.022), and “middle” and “far-right” (*P* = 0.026). There was a trend toward a significant difference between “middle” and “left” positions (*P* = 0.055). All other differences were not significant (*P* ≥ 0.150).

In *experiment 4*, there was a significant difference between “far-low” and “middle” (*P* = 0.016), between “far-low” and “high” (*P* = 0.001), between “far-low” and “far-high” (*P* < 0.0005), and between “low” and “far-high” (*P* = 0.039) positions. All other differences were not significant (*P* ≥ 0.087).

In *experiment 5*, there was a significant difference between “front” and “rotated side” (*P* = 0.037), between “front” and “side” (*P* = 0.047), between “front” and “rear” (*P* = 0.002), between “rotated front” and “rotated side” (*P* = 0.038), between “rotated front” and “side” (*P* = 0.045), and between “rotated front” and “rear” (*P* = 0.032). All other differences were not significant (*P* ≥ 0.697).

In *experiment 6*, there was a significant difference between “middle” and all other positions: “middle” and “far-low” (*P* = 0.047), “middle” and “rotated side” (*P* = 0.015), “middle” and “far-right” (*P* = 0.027), and “middle” and “ultra-far” (*P* = 0.027). All other differences were not significant (*P* ≥ 0.285).

### Model Fitting

**Fig. 3.**
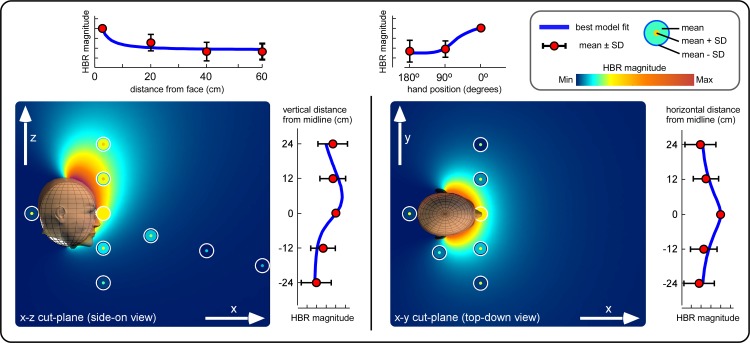
Effect of hand position on HBR magnitude and geometric modeling of DPPS: combined description of the experimental data with the best-fitting geometric model. The measured HBR data (mean ± SD) are represented as concentric circles located where the measurements were taken. The background color represents the HBR magnitude predicted by the best-fitting geometric model. The line graphs at the side of each color plot show HBR magnitudes (mean ± SD) along each axis, together with the best-fitting geometric model (blue line). The best-fitting model indicates that the DPPS extends from the face area and has the shape of an elongated bubble, extending forward and upward.

Figure 3 shows the magnitude of the HBR elicited when the hand was in different positions from all six experiments. It also shows how the best-fitting geometric model compares to the measured data. The GoF and parameter values of all considered models are summarized in Table 1.

**Table 1.** Goodness of fit and gravity parameter of each geometric model

Geometric Shape	Corresponding Body Part	GoF	*P* Value	*C*_grav_
Full ellipsoid	Head	7.595	<0.0001	−1.1
Half-ellipsoid	Face	**0.1832**	**0.3742**	−1.05
Quarter-ellipsoid	Half-face	1.944	0.044	−0.1
Two half-ellipsoids	Eyes	**0.850**	**0.1831**	−0.85

GoF, goodness of fit; *C*_grav_, gravity parameter.

GoF < 1.850 and *P* > 0.05 indicate a significant fit. Significant values are in boldface.

The geometric model that best fitted the data was the half-ellipsoid (*model 2*: *P* = 0.3742, GoF = 0.1832, *C*_grav_ = 1.05). It predicted a DPPS with a pseudoellipsoidal shape, which extends from the face upward and outward (Fig. 3). Therefore, the modeled DPPS was symmetric on the horizontal plane but asymmetric on the vertical plane with respect to the center of the face. Note that a *P* > 0.05 indicates that the model is accepted, and the larger the *P* value, the better the model fit. Concurrently, the lower the GoF, the better the model fit.

The only other model to fit the data significantly was the eyes-only model (*model 4*: *P* = 0.1831, GoF = 0.850, *C*_grav_ = 0.85), which predicted a DPPS qualitatively similar to that described above at the distances tested, albeit with two distinct areas of further increase in predicted HBR magnitude, one around each eye.

The models in which the defended area was represented by a full ellipsoid (*model 1*: corresponding to the whole head) and a quarter-ellipsoid (*model 3*: corresponding to the top half of the face) were both rejected (*model 1*: *P* < 0.0001, GoF = 7.595; *model 3*: *P* = 0.044, GoF = 1.944).

Both accepted models predicted that the HBR increases monotonically and nonlinearly with the proximity between the threat and the defended area. Given that the *P* values of *models 2* and *4* were >0.05 (a threshold value that, within the χ^2^ distribution considered, corresponds to a GoF of 1.850), these models were accepted by the data, thus indicating that three parameters (*a*, *b*, and *C*_grav_) are sufficient to significantly fit the data from all six experiments.

## DISCUSSION

In this study we developed a family of theoretical models to characterize the fine-grained topography of the DPPS, defined by the HBR increase, as a function of the spatial position of the hand in egocentric coordinates. These models are based on a set of assumptions about how the nervous system represents threatening stimuli. We mathematically expressed the strength of the defensive HBR as a function of the probability of the face being hit by a threat, which in turn is a function of the position of the stimulated hand in space. We performed a combined analysis of two previous data sets and four newly collected data sets. We obtained four main results.

First, the HBR magnitude is affected by the position of the hand on the coronal plane along both a medio-lateral and a vertical axis (Fig. 3)—that is, stimuli delivered in the “middle” position elicited significantly stronger responses than stimuli delivered in more lateral (*experiment 3*) and lower (*experiment 4*) positions. Second, the HBR magnitude is not increased when the hand is behind the head, even if the hand is very near to the head (*experiment 5*). Third, the HBR magnitude is modulated by the position of the hand in head-centered coordinates (*experiment 5*). The fourth result is derived from the previous three. Geometric modeling revealed that the DPPS defined by the HBR has the shape of an elongated bubble, extending forward and upward from the face (Figs. 2 and 3). Defensive responses to stimuli located within this area increase monotonically in a nonlinear fashion.

### 

#### HBR modulation by threat position on the coronal plane.

The modulation of HBR magnitude as a function of the egocentric position of the threat has so far only been explored along the sagittal plane (Sambo et al. 2012a, 2012b; Sambo and Iannetti 2013). Along that plane, the HBR magnitude increases monotonically and nonlinearly with proximity of the hand to the face. This result allowed us to infer the extent of the DPPS in front of the face but did not provide any information on the shape of the DPPS. Here we added two spatial dimensions to the DPPS characterization and measured the HBR magnitude along a medio-lateral axis (*experiment 3*) and a vertical axis (*experiment 4*) along the coronal plane. We found that the stimulation of the hand in the “middle” position elicited a significantly stronger HBR than in the lateral and inferior positions. Therefore, the HBR magnitude increased with proximity of the hand to the face even when the position of the threat was displaced perpendicularly to the previously explored axis. This indicates that the increase in HBR magnitude with proximity of the hand to the face is consistent across different spatial dimensions. This experimental observation is in accordance with the qualitative concept of the DPPS as an area within which potentially harmful stimuli pose a greater threat, and thus elicit stronger defensive reactions.

The dependence of behavioral and physiological responses on the position of the stimuli in egocentric coordinates is also supported by a range of measures other than the HBR, regardless of whether the response is goal oriented or threatening (for a discussion on different types of peripersonal space, see de Vignemont and Iannetti 2015). For example, multisensory integration of stimuli belonging to different sensory modalities varies as a function of the proximity between the stimulus and the body (i.e., Ladavas 2002; Longo and Lourenco 2006, 2007; Occelli et al. 2011). Similarly, proximity shortens reaction times to innocuous stimuli within the peripersonal space (Valdés-Conroy et al. 2014) and enhances the galvanic skin response elicited by a threatening stimulus (Combe and Fujii 2011).

The results of *experiments 3–6* provide novel physiological data, as they describe the HBR modulation along different spatial dimensions and therefore roughly describe the shape of the DPPS. Although the HBR increase seems overall consistent in different spatial dimensions, always increasing monotonically when the distance between the threat and the face was reduced, we observed some interesting differences in the HBR modulation along the rostro-caudal axis on the coronal plane. Indeed, out of all stimulus positions used in *experiment 4* (“far-low,” “low,” “middle,” “high,” “far-high”) the HBR magnitude at the “middle” position was only significantly different from that at the “far-low” position. Therefore, along this axis the DPPS does not seem to expand symmetrically from the center of the face, as it does along the medio-lateral direction (Fig. 3).

#### Absence of HBR modulation when the threat is behind the head.

In *experiment 5* we observed that placing the threat in rear space, i.e., directly behind the head, did not increase the HBR magnitude (the HBR magnitude at the “rear” position was not different from that at either of the “side” conditions but was significantly smaller than the HBR magnitude at the “front” conditions; Fig. 3). At first glance, this result might seem at odds with the observation that audio-tactile interactions, which are commonly used as an indicator of peripersonal space, are stronger in rear space than in frontal space (e.g., Farnè and Làdavas 2002). However, the different modulations of audio-tactile and HBR measures in rear space are entirely compatible and depend on the functional significance of the physiological measure chosen. Indeed, the lack of visual representation in rear space forces individuals to rely more on auditory processing to detect threats behind the head (Occelli et al. 2011; Van der Stoep et al. 2014). Furthermore, the audio-tactile measures reflect the detection of general bodily threats, while the HBR is a defensive response spatially related to the eye. Consequently, the HBR is only expected to be modulated when the threat is located in a subset of the positions within which audio-tactile interactions are modulated.

#### HBR modulation depends on hand position in a head-centered reference frame.

In *experiment 5*, we also demonstrated that the HBR modulation depends on hand position in a head-centered reference frame: rotating the head altered the HBR magnitude when the hand was kept in the same position but not when the hand moved along with the head. Indeed, we found no difference in HBR magnitude between the “side” and “rotated side” or between the “front” and “rotated front” conditions (Fig. 3). This result supports the notion that the DPPS is coded in body part-centered frames of reference (Graziano and Cooke 2006).

#### A geometric model underlying the increase in HBR.

The major objective of this study was to develop a family of geometric models that describe the DPPS defined by the HBR, by modeling the dependence of the HBR magnitude on the respective position of the threat and the body district to be defended. These geometric models describe the spatial dependence between the magnitude of defensive responses and the position of the threat in peripersonal space. Notably, in these models, a predicted HBR magnitude is defined for all points in space. Therefore, to determine the shape of the DPPS, one must define a cutoff value and consider all positions at which the predicted HBR magnitude is larger than the cutoff to be inside the DPPS. Importantly, regardless of the chosen cutoff value, the shape of the volume described by the best-fitting model was an elongated bubble, extending mainly from the top half of the face (Figs. 2 and 3). This bubblelike shape was symmetric on the horizontal plane (i.e., it extended equally on the right and left sides) but asymmetric on the vertical plane (i.e., it extended more above than below the center of the face). Within this bubble, the defensive response increases monotonically and nonlinearly with proximity to the defended area.

The geometries tested here were not exhaustive of all possible geometries of defended body areas. We could, in fact, have tested an infinite range of geometries, representing every body part. However, because the blink reflex consists of the contraction of eye-closing muscles, we expect the modulation of HBR magnitude to reflect how the nervous system represents the defensive space surrounding the face and/or the head (rather than, for example, the back or the foot). In fact, from the results of *experiments 3–5* it can be intuitively seen that geometries not reflecting areas on or around the head would not fit the data. Therefore, we limited our analysis to a range of shapes representing parts of the head, which were most likely to give strong-fitting results.

The choice of using a set of geometric models to study the spatial features of the DPPS was driven by three lines of reasoning.

First, such models can predict the magnitude of defensive responses to sensory events at all locations within the peripersonal space (i.e., at locations that have not been experimentally measured). Notably, although other mathematical functions (e.g., a fitted polynomial) could achieve a similar prediction, they are not explicative of the underlying physiological principle explaining the observed HBR modulation.

This is the second reason why we chose to use geometric models: they arise from a set of physiological assumptions about the rules the nervous system obeys to regulate the magnitude of a defensive response. In other words, these models allow for the testing of the physiological assumptions of why the HBR increases as a function of hand position. The assumptions we defined imply that the nervous system calculates the probability that a body part is harmed by a potentially dangerous stimulus. This calculation takes into account *1*) stimulus position, *2*) the shape of the defended area, and *3*) the effects of gravity. Based on the calculated harm probability, the nervous system consequently adjusts the strength of the defensive action. Therefore, the observed best fit of geometric *model 2* (Fig. 3) provides physiological information about how the body area to be defended and the surrounding DPPS are represented: specifically, it supports the idea that the nervous system regulates the excitability of the circuitry underlying the HBR to defend the face in particular. However, the solid fit of *model 4* (Fig. 2) does not allow us to exclude the possibility that the HBR is modulated to defend the eyes. Given that the area with the largest difference between the HBR magnitudes predicted by the two models is a small region at the nasion (Fig. 2), it is practically extremely difficult to measure the HBR at that position. Therefore, the small differences in HBR magnitude at the tested positions were not sufficient to reject either model. Importantly, the DPPS shapes resulting from the two models are very similar (Fig. 2).

Further physiological information comes from the nonzero value of the optimized variable *C*_grav_. This observation suggests that the nervous system takes gravity into account when estimating the probability of being hit by a threat. This is consistent with existing evidence that humans and nonhuman primates have internal models to estimate the effects of gravity, in relation both to one's own posture (Angelaki et al. 2004; Merfeld et al. 1999) and to external objects (Schwartz 1999).

The third reason for choosing a geometric model is that they can be generalized to describe a range of potentially defensive responses, as long as the assumptions on which the models are based hold true. Indeed, the framework of the geometric models described in this study can be applied to any shape of defended area, any type of defensive response, or any type of eliciting stimulus. For example, the geometric properties of the shortening of response times to tactile stimuli as a function of the relative position between auditory or visual stimuli and the hand (Macaluso and Maravita 2010; Occelli et al. 2011; Sambo and Forster 2009; Serino et al. 2007) would be amenable to formal investigation with the approach described here, as would the modulation of the activity of bimodal visuotactile neurons as a function of the location of a visual stimulus (Fogassi et al. 1996; Graziano and Cooke 2006).

It is interesting to note that the tactile receptive field of such bimodal visuotactile neurons could be seen as a counterpart of the concept of a defended area presented in this article. Similarly, the visual receptive field of such neurons (which surrounds and is anchored to their tactile receptive field; Fogassi et al. 1996) could be seen as a counterpart of the concept of a DPPS anchored to the area to defend. In this way, the geometric model can be seen as a formalized bridge between low-level physiological data (i.e., firing rates of bimodal parietal neurons; Graziano and Cooke 2006) and higher-level defensive behaviors (i.e., blinking; Sambo et al. 2012b).

## GRANTS

G. D. Iannetti acknowledges the generous support of The Wellcome Trust. R. J. Bufacchi is supported by the Engineering and Physical Sciences Research Council (EPSRC) and the Medical Research Council (MRC) through UCL CoMPLEX (http://www.ucl.ac.uk/complex).

## DISCLOSURES

No conflicts of interest, financial or otherwise, are declared by the author(s).

## AUTHOR CONTRIBUTIONS

Author contributions: R.J.B. and G.D.I. conception and design of research; R.J.B. performed experiments; R.J.B., M.L., and L.D.G. analyzed data; R.J.B., L.D.G., and G.D.I. interpreted results of experiments; R.J.B. and G.D.I. prepared figures; R.J.B. and G.D.I. drafted manuscript; R.J.B., M.L., L.D.G., and G.D.I. edited and revised manuscript; R.J.B., M.L., L.D.G., and G.D.I. approved final version of manuscript.
